# Novel blends of polylactide with ethylene glycol derivatives of POSS

**DOI:** 10.1007/s00396-014-3344-3

**Published:** 2014-09-10

**Authors:** Anna Zubrowska, Ewa Piorkowska, Anna Kowalewska, Michal Cichorek

**Affiliations:** Centre of Molecular and Macromolecular Studies, Polish Academy of Sciences, Sienkiewicza 112, 90-363 Lodz, Poland

**Keywords:** Polylactide, Poly(ethylene glycol), Polyhedral oligomeric silsesquioxanes, Toughening

## Abstract

Polylactide (PLA), a main biodegradable and biobased candidate for the replacement of petrochemical polymers, is stiff and brittle at room conditions. It is therefore of high interest to formulate new PLA-based materials suitable for applications demanding flexibility and toughness. In this work, novel blends of PLA with polyhedral oligomeric silsesquioxanes (POSS) grafted with longer (P1) and shorter (P2) arms of ethylene glycol derivatives were prepared and studied. It was hypothesized that, owing to their architecture with the central POSS cage grafted with arms, miscibility and stability of the blends could be improved. Indeed, PLA/P1 blends were homogeneous despite P1 relatively high *M*_w_ of 9,500 g mol^−1^. The blend with 20 wt% of P1, having *T*_g_ at 16 °C, was transparent and flexible, elastomer-like material with excellent drawability. The blend remained homogeneous and retained its good drawability as well as flexibility during 6 months of aging at room temperature: a 2 % secant modulus of elasticity well below 100 MPa, a low yield stress below 2 MPa, and and a large strain at break of 8 (800 %). Contary to that, PLA/P2 blends were only partially miscible. Nevertheless, owing to the liquid state of the dispersed phase, the blend with 15 wt% of P2 was transparent and ductile, with *T*_g_ at 49 °C, a relatively high yield strength of 29 MPa, and a large strain at break of 2.3 (230 %). The toughening mechanism involved the initiation of crazes and facilitation of their propagation by the liquid inclusions via the local plasticization effect.

## Introduction

Increasingly important polylactide (PLA), a main biodegradable and biobased candidate for replacement of petrochemical polymers, which can be produced from annually renewable resources, has the glass transition temperature *T*_g_ of 55–60 °C. As a consequence, PLA is stiff and brittle at ambient conditions, which limits its applications demanding high toughness and drawability. The ability of PLA to crystallize depends on its enantiomeric composition. Both optically pure poly(l-lactide) and poly(d-lactide) are crystallizable polymers, but a decrease of the optical purity lowers crystallizability of PLA. Slowly crystallizing PLAs could be quenched below *T*_g_ without crystallization and cold-crystallized during heating from the glassy state [1]. Crystallinity, if developed, increases slightly the stiffness but further decreases the drawability of PLA [2].

To improve its flexibility and ductility, PLA has been plasticized with various plasticizers of low and high molar mass (*M*), including citrate esters [3, 4], triacetine [4], poly(ethylene glycol) (PEG) [5–11], and poly(propylene glycol) [12, 13], block copolymers of ethylene glycol and propylene glycol [14], poly(ethylene adipate) and poly(diethylene adipate) [15], diethyl bishydroxymethyl malonate oligoester and oligoesteramide [16, 17]. By increasing PLA's chain mobility, plasticization decreases *T*_g_, yield stress and elastic modulus, and improves elongation at break. Apart from the desired mechanical properties, there are other requirements like non-volatility and biodegradability of a plasticizer. Since PEG fulfils that prerequisite, plasticization of PLA with PEG was widely investigated in the past [e.g., 8–11]. However, the key aspect is stability of plasticized PLA. Owing to their high mobility, plasticizers with low *M* migrate within PLA matrix. Even PEG with *M* of 1,000 g mol^−1^ migrated from the bulk of plasticized PLA and accumulated on its surface [18]. The use of PEGs with even higher molar masses creates other problems—phase separation and crystallization of PEG in PLA/PEG blends can occur in a degree dependent on concentration and also on *M* of PEG [8–10, 18]. Hence, although plasticization led to improvement of flexibility and ductility of PLA, it usually did not allow reaching low modulus elastomeric-like behavior stable over time. For instance, Hu et al. [9, 10] demonstrated that unaged PLA/PEG blends, containing 30 wt% of PEG with *M* of 8,000 g mol^−1^, were low modulus elastomeric-like materials with *T*_g_ at 7 °C, but they aged via either crystallization of PEG and PLA or phase separation and, as a result, their *T*_g_ increased and behavior changed over a relatively short time to higher modulus thermoplastic-like. After 30 h, the yield stress and 2 % secant modulus of elasticity of the blend reached 2 and 100 MPa, respectively [9] whereas, after 75 days, these values further enlarged four times and *T*_g_ increased to 27 °C from 7 °C.

Recently, grafting PEG on maleated PLA chains was explored to improve stability and compatibility between the components [19]. However, the elastomer-like behavior was not reached; the blend with 20 wt% of PEG exhibited a relatively high yield stress of nearly 16 MPa, even though tested at a low rate of 0.06 min^−1^. Although grafting of tributyl citrate on maleated PLA permitted reaching a yield stress of about 7 MPa, after 6 months of aging, dynamic mechanical thermal analysis evidenced an increase of *T*_g_ and signs of phase separation in this blend [20].

Plasticization is unavoidably associated with a decrease of *T*_g_, yield strength, and stiffness. Toughening of PLA by blending with immiscible polymers [e.g., 21–23] allows maintaining its *T*_g_ and diminishes less the yield strength and elastic modulus. It is long recognized that multiple crazing initiated by dispersed rubber phase is one of the main toughening mechanisms acting in systems with a glassy matrices, for instance, in high-impact polystyrene [24]. The second well-known phenomenon is cavitation in dispersed inclusions of the minor component, which facilitates shear yielding of the glassy matrix [e.g., 25]. In PLA-based blends, the cavitation either inside the inclusions [22] or at the inclusion–matrix interface [21] was observed. It was also demonstrated [22] that the poly(1,4-*cis*-isoprene) particles dispersed in PLA initiated crazes, but cavitation inside the particles promoted the change of deformation mechanism of matrix to shear yielding.

In this study, in order to obtain stable PLA-based ductile materials, PLA was melt-blended with hybrids of ethylene glycol derivatives and polyhedral oligomeric silsesquioxane (POSS). POSSs are a relatively new class of materials, which are used for modification of polymers. POSS molecules have the general formula R_*n*_(SiO_1.5_)_*n*_, where R can be hydrogen, an organic group or polymer chain; for the most common octameric structure, *n* = 8. POSS cages grafted with polymer chains are multiarm polymers, which can exhibit properties and miscibility with other polymers different from their linear counterparts [e.g., 26, 27]. Moreover, although the thermal properties of such hybrids are chain-length-dependent, they can have lower *T*_g_s than their linear analogs and decreased, or even entirely suppressed, crystallizability [27]. We hypothesized that blending PLA with multiarm polymers obtained by grafting of PEO or PEG arms on POSSs would allow obtaining a ductile material stable over time.

Telechelic PEG–POSS polymers can be obtained through formation of direct urethane linkage between hydroxy end groups of PEG and isocyanate groups of POSS macromer [28, 29]. PEG-substituted octasilsesquioxanes, prepared by the hydrosilylation of unsaturated ethylene glycol monoallyl ethers (allyl-PEG) of various chain lengths (two to six repeating units) with both octakis(dimethylsiloxy)octasilsesquioxane and octahydridosilsesquioxane, were also reported [30]. The respective allyl ethers can be synthesized through the reaction of hydroxyl end groups with allyl bromide in the presence of a base (NaOH [27, 31–33], NaH [34], or alkali metals [30, 35]).

In general, POSS is non-biodegradable and, owing to its hydrophobic nature and inertness to hydrolysis, can slow down hydrolytic degradation of a material in which it is dispersed [e.g., 36]. However, POSS has been demonstrated to be nontoxic and cytocompatible [37, 38]. Recently, the use of PEG–POSS in biomedical applications was explored, for instance for drug delivery [39] or to obtain hydrogels designed for scaffolds for bone repair [40].

In the present study for modification of PLA two POSS hybrids were used, with shorter and longer arms, being derivatives of ethylene glycol. In order to avoid crystallization-induced phase separation, PLA with low stereoregularity, containing 18 wt% of d-lactide, was used. Both types of hybrids efficiently modified the mechanical properties of PLA. Especially PEG methyl ether grafted POSS exhibited very good miscibility with PLA, despite its relatively large molar mass of 9,500 g mol^−1^ and acted as an efficient plasticizer, which allowed us to obtain transparent, elastomer-like low modulus material with excellent drawability retaining its good properties during 6 months.

## Experimental

The study utilized PLA 4060D purchased from NatureWorks LLC (Minnetonka, MN), with density of 1.24 g cm^−3^, weight average molar mass *M*_w_ of 120 kg mol^−1^ and polydispersity *M*_w_ *M*_n_^−1^ = 1.4 as determined by size exclusion chromatography (SEC)with multi-angle laser light scattering detector in dichloromethane. d-Lactide and l-lactide contents were 18 and 82 mol%, respectively, as determined by measurements of specific optical rotation.

Two different POSS hybrids were used. R_*n*_(SiO_1.5_)_*n*_ with R = CH_2_CH_2_(OCH_2_CH_2_)_m_OCH_3_, being a cage mixture with *n* of 8, 10, 12 and average *m* of 13.3 (P1), was purchased from Hybrid Plastics Inc. (Hattiesburg, MS). According to the supplier, density and PEG content were 1.2 g cm^−3^ and 92 wt%, respectively. Molar mass of a single arm calculated based on *m* of 13.3 was equal to 644.2 g mol^−1^. *M*_w_ and *M*_w_ *M*_n_^−1^ of P1 were 9,500 g mol^−1^ and 1.3, respectively, as determined by a SEC method in aqueous solution, with triple detection (LDC RI detector and Viscotek T60A dual detector) on the chromatograph (Knauer K-501 HPLC pump) with a set of TSK-GEL columns (G5000 PW_XL_ + 3000 PW_XL_ + 2500 PW_XL_) at 26 °C.

R_8_(SiO_1.5_)_8_ with R = OSi(CH_3_)_2_CH_2_CH_2_CH_2_(OCH_2_CH_2_)_2_OCH_2_CH_3_ (P2) was synthesized by hydrosilylation of 3-[2-(2-ethoxyethoxy)ethoxy]-propene-1 with octakis(dimethylsiloxy)octasilsesquioxane [HSi(CH_3_)_2_O]_8_(SiO_1.5_)_8_. Monoallyl ether of di(ethylene glycol) ethyl ether was obtained according to the slightly modified literature procedure [32]. The reaction with allyl bromide was exothermic, and there was no need to increase the temperature after the addition of allyl bromide. The hydrosilylation reaction was carried out in the presence of a platinum catalyst (Karstedt’s catalyst). The stoichiometry of the reaction was controlled in order to obtain octasubstituted POSS and not to leave any remnant of unreacted 3-[2-(2-ethoxyethoxy)ethoxy]-propene-1 in the reaction mixture. Both the formed 3-[2-(2-ethoxyethoxy) ethoxy]-propene-1 and octasubstituted product were characterized by ^1^H NMR spectroscopy. The resonance signals in ^1^H NMR spectrum were attributed to fragments of side groups, and the number of repetitive glycolic units was estimated. *M* of octakis{3-[2-(2-ethoxyethoxy)ethoxy]-propyldimethylsiloxy}-octasilsesquioxane, calculated on the basis of ^1^H NMR spectrum was 2,409 g mol^−1^, whereas that of a single arm was 249 g mol^−1^. For clarity, further details of the synthesis of P2 are given in the Appendix.

Both POSS hybrids were liquid at room temperature. *T*_g_s of P1 and P2 measured by differential scanning calorimetry (DSC) during heating at 10 °C min^−1^ were at −81 °C and −84 °C, respectively. Melting peak, at −2 °C, was observed only for P1. The melting enthalpy was 76 J g^−1^, which corresponded to PEG crystallinity level of 56 wt% for the PEG content of 92 wt% and melting enthalpy for 100 % crystalline PEG of 146.7 J g^−1^ [41]. During cooling P1 crystallized at −26 °C.

Prior to blending, the components were vacuum-dried for 4 h: PLA at 100 °C and both POSS hybrids at 90 °C. Melt-blends containing from 5 to 20 wt% of POSS (selected compositions) were prepared using a Brabender batch mixer (Duisburg, Germany) operating at 180–190 °C for 10 min at 60 rpm, under the flow of dry gaseous nitrogen. Neat PLA was also processed under the same conditions to obtain a reference material. The blends will be referred to as, for example, PLA/P1-15, where 15 stands for the P1 content in weight percent.

The 0.5- and 1-mm-thick films of all the materials were prepared by compression molding at 180 °C for 3 min in a hydraulic hot press followed by quenching between thick metal blocks kept at 15 °C. The films were then stored in dry atmosphere (relative humidity of 10 %) in desiccators at room temperature. The thermal and mechanical properties of the films were tested within 7 days. Films of PLA/P1-20 were stored for 6 months, and their properties were examined over this period of time. In addition, specimens of this blend were held for 4 weeks at 35 °C in a sand bath in order to determine the influence of aging at elevated temperature on their thermal properties.

The films were examined by DSC carried out with a TA Instrument 2920 DSC (New Castle, DE) at a heating rate of 10 °C min^−1^. *T*_g_ was taken as a temperature corresponding to the midpoint of the heat capacity increment. Aged PLA/P1-20 after the first heating to 195 °C was cooled to zero Celsius and heated again at 10 °C min^−1^.

Dynamic mechanical thermal analysis (DMTA) was carried out in a dual-cantilever bending mode with a DMTA Mk III, Rheometric Scientific Ltd. apparatus (Epsom, UK) at a frequency of 1 Hz and a heating rate of 2 °C min^−1^ on rectangular samples, 10 mm × 30 mm, cut from 1-mm-thick films.

For tensile tests, oar-shaped specimens conforming to ISO 527–2, with a gauge length of 25 mm and a gauge width of 5 mm, were cut from 0.5-mm-thick films. Uniaxial stress–strain measurements were performed on an Instron tensile testing machine (High Wycombe, UK) at a rate of 0.4 min^−1^ (40 % min^−1^) at 25 °C in a temperature chamber with circulating air. At least three specimens of each material were tested.

To have an insight into the structure, cryo-fracture surfaces of the blends were studied under a Jeol 5500LV and Jeol 6010LA (Tokyo, Japan) scanning electron microscopes (SEM) after sputtering with gold. In addition, the blends were cryo-microtomed to produce specimens with flat and smooth surfaces, which were coated with carbon using electric arc spraying, and analyzed by SEM with energy-dispersive spectroscopy (EDS). Selected specimens were examined also by polarized light microscopy (PLM).

## Results and discussion

### Characterization of the blends

**Fig. 1:**
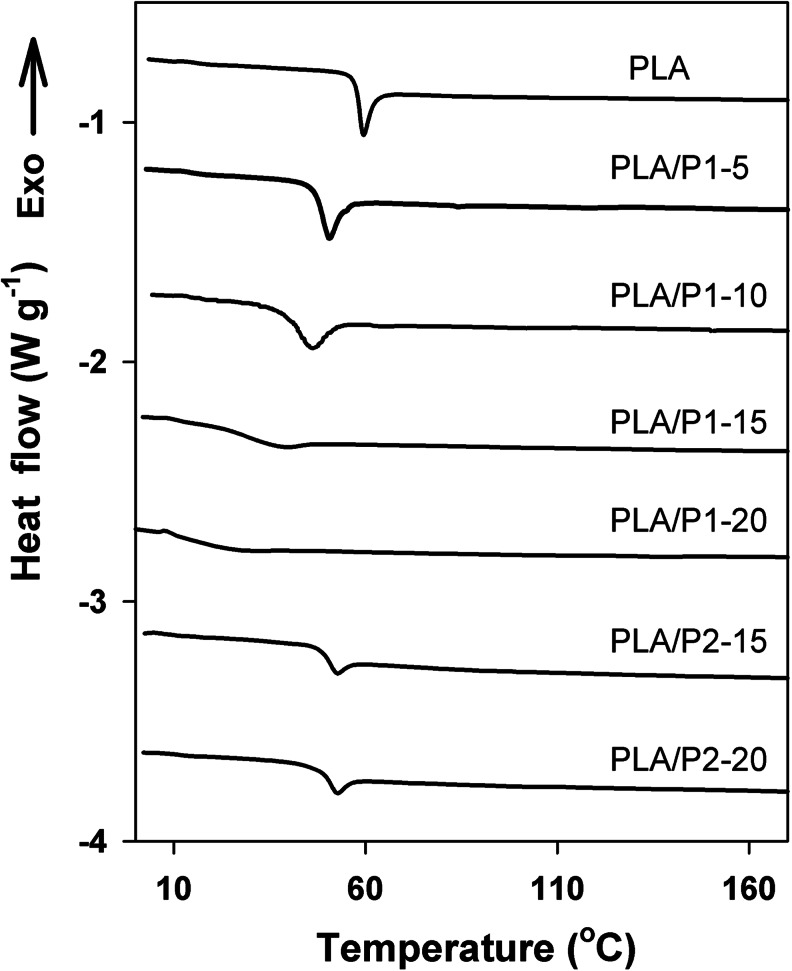
DSC heating thermograms of PLA, PLA/P1, and PLA/P2 blends. Heating rate of 10 °C min^−1^. Thermograms shifted vertically for clarity

The heating thermograms of the quenched films of the PLA and PLA-based blends are collected in Fig. 1, whereas their *T*_g_s determined from DSC are listed in Table 1. The heating thermograms evidenced that PLA in all the materials was amorphous and unable to cold-crystallize during heating at 10 °C min^−1^. All the blends exhibited *T*_g_s below that of neat PLA, 57 °C. While for PLA/P2 blends the decrease of *T*_g_ was rather moderate, by 8–9 °C for both compositions, *T*_g_ of PLA/P1 blends decreased gradually with increasing P1 content and reached 16 °C for PLA/P1-20. No evidence of melting of P1 in the blends was detected, indicating that it did not form a separate phase.

**Table 1:** *T*_g_ of PLA, PLA/P1, and PLA/P2 blends: *T*_DSC_ measured by a DSC method and temperatures of loss modulus *E*″ peaks, *T*_1E″_, and *T*_2E″_

Sample code	*T*_DSC_ (°C)	*T*_1E″_ (°C)	*T*_2E″_ (°C)
PLA	57	–	55
PLA/P1-5	48	–	49
PLA/P1-10	39	–	39
PLA/P1-15	29	–	30
PLA/P1-20	16	–	16
PLA/P2-15	49	−77	50
PLA/P2-20	48	−79	49

**Fig. 2:**
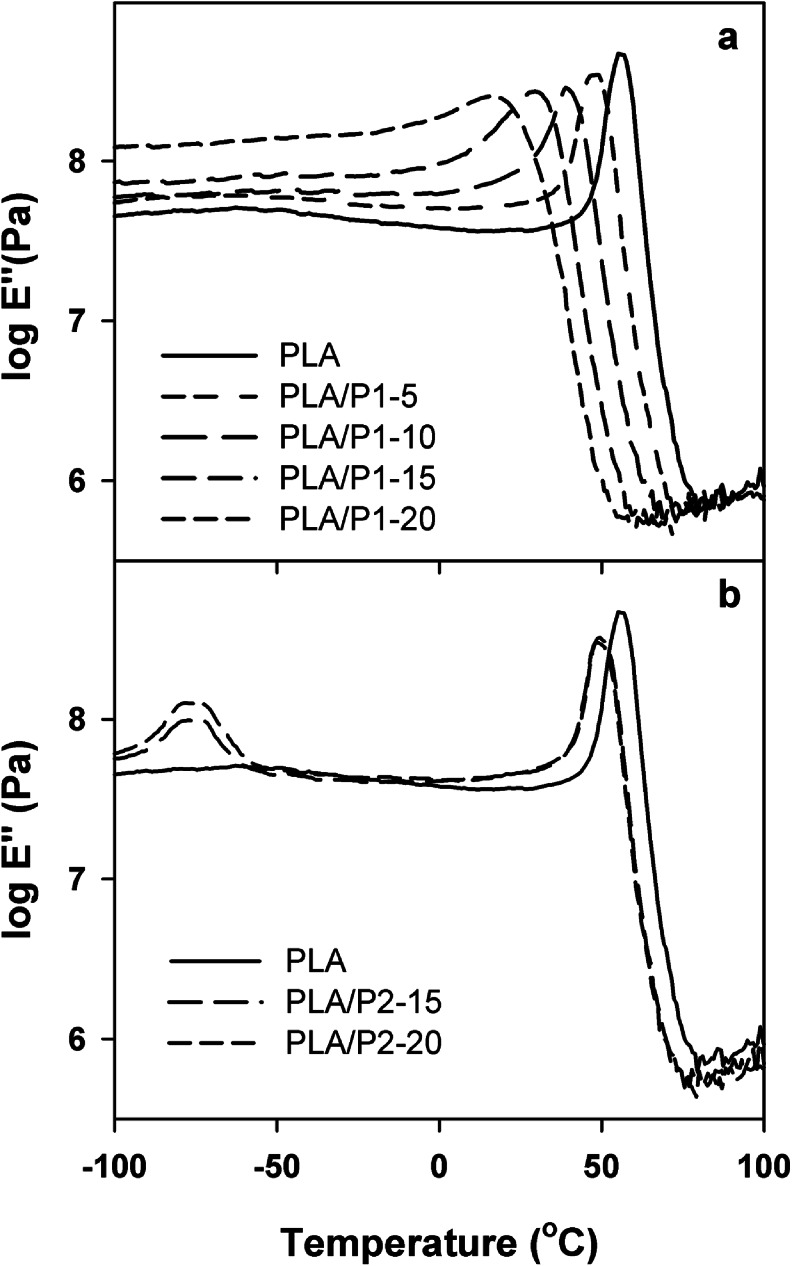
Temperature dependencies of loss modulus *E*'' of PLA, PLA/P1 (**a**), and PLA/P2 (**b**) blends

**Fig. 3:**
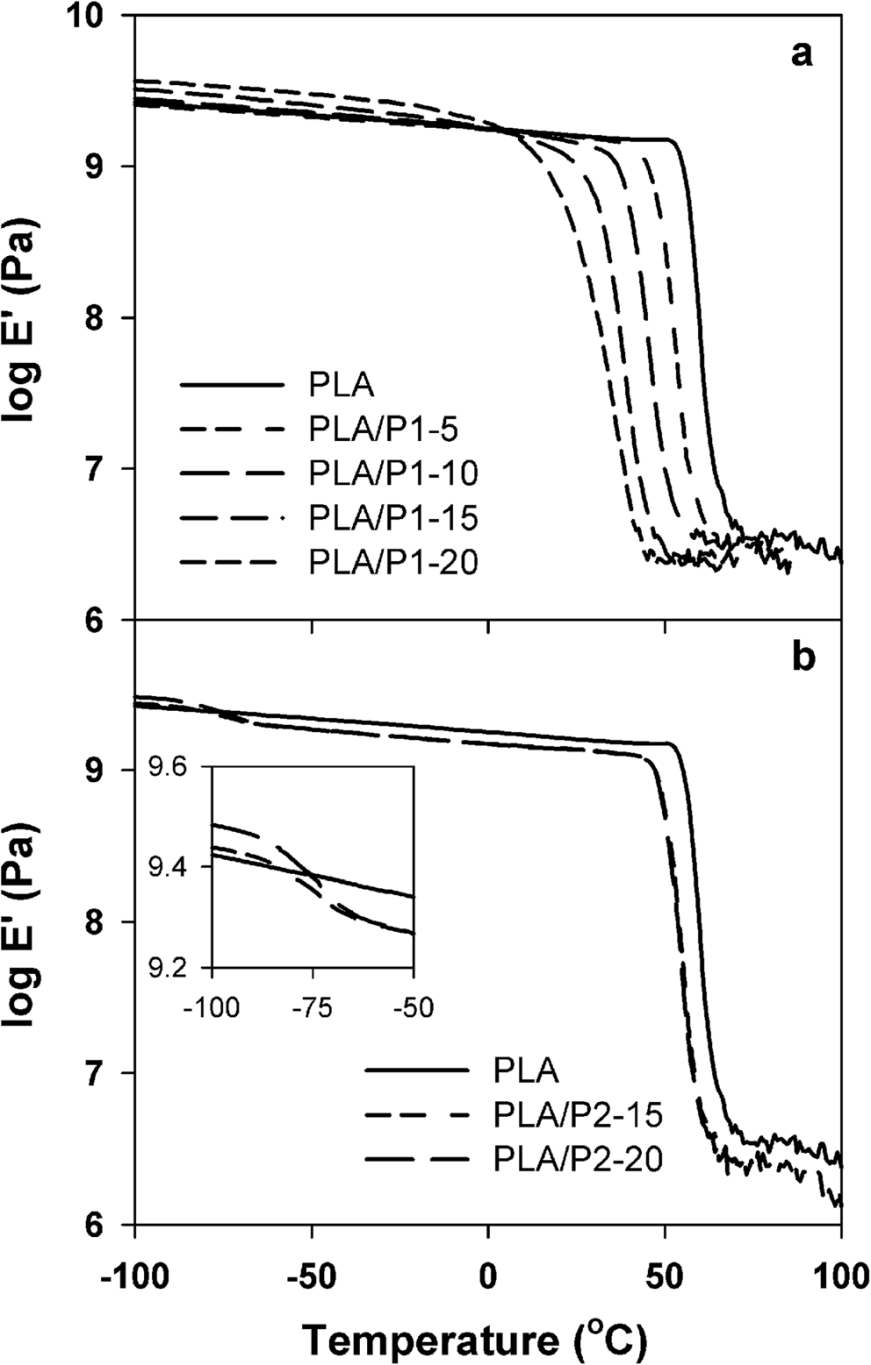
Temperature dependencies of storage modulus *E*' of PLA, PLA/P1 (**a**), and PLA/P2 (**b**) blends

Temperature dependencies of loss modulus *E*'' and storage modulus *E*' of the materials are compared in Figs. 2 and 3. The *E*″ peak temperatures, which are listed in Table 1, correlated closely with *T*_g_s from DSC. Neat PLA exhibited a single *E*'' peak at 55 °C. The *E*'' temperature dependencies of PLA/P1 blends were also featured by single peaks, but the peak temperature decreased with increasing P1 content, from 49 °C for PLA/P1-5 to 16 °C for PLA/P1-20, which evidenced good miscibility of the components and plasticizing effect of P1 on PLA. In addition, the *E*'' peaks of PLA/P1 widened with increasing P1 content reflecting broadening of the spectrum of relaxation times; *E*'' of PLA/P1-20 started to rise even below zero Celsius. Contrary to that, the two separate *E*'' peaks indicated phase separation in PLA/P2 blends. The *E*'' peaks at −77 and −79 °C reflected the glass transitions in P2-rich phase whereas those at about 50 °C corresponded to the glass transition in the PLA-rich phase. The temperature of the latter, below *T*_g_ of neat PLA, indicated partial miscibility of the components.

*E*' of neat PLA behaved in a typical way, decreasing with increasing temperature and falling below 10 MPa in the temperature range of glass transition. Below zero Celsius, *E*' of PLA/P1 blends was close to that of neat PLA for small contents of P1 but enlarged with increasing content of the modifier. *E*' of each blend diminished with increasing temperature and finally dropped rapidly in the glass transition temperature range. As a result, from 10 to 70 °C *E*' strongly decreased with increasing P1 content and was the smallest for PLA/P1-20.

Below −90 °C *E*' of PLA/P2-15 and especially of PLA/P2-20 exceeded *E*' of neat PLA, but, in the range of low-temperature glass transition, from approximately −90 °C to approximately −60 °C, it dropped below that of PLA. The second and much more pronounced rapid fall of *E*' occurred in the temperature range of glass transition in the PLA-rich phase of the blends.

**Fig. 4:**
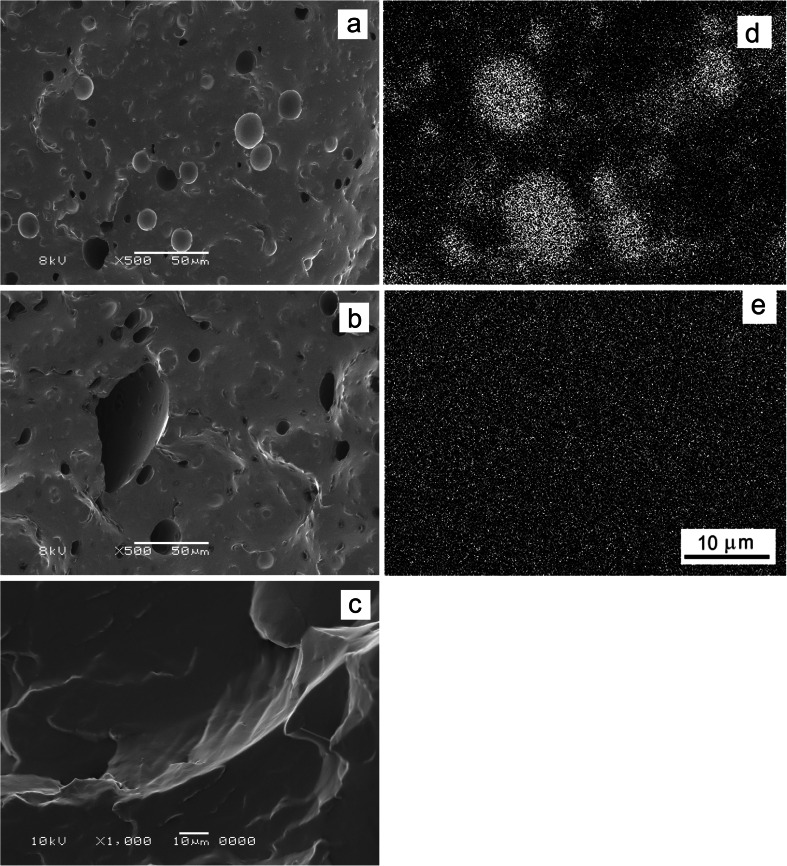
SEM micrographs of cryo-fracture surfaces of blends: PLA/P2-15 (**a**), PLA/P2-20 (**b**), PLA/P1-20 (**c**), and X-ray Si mapping of: PLA/P2-15 (**d**), PLA/P1-20 (**e**). Magnification in (**d**) the same as in (**e**)

All PLA/P1 and PLA/P2 blends were transparent. Nevertheless, SEM examination of cryo-fracture surfaces confirmed the phase separation in the PLA/P2 blends. Distinct holes where P2 was accumulated are clearly visible on micrographs in Fig. 4a and b. The inclusions were larger in PLA/P2-20 than in PLA/P2-15 with maximum size of 90 and 30 μm, respectively. Contrary to that, no evidence of the phase separation was found in the PLA/P1 blends, even at the highest P1 content of 20 wt%, as shown in Fig. 4c. These findings were confirmed by EDS analysis as shown in Figs. 4d and e, which present Si mapping.

**Fig. 5:**
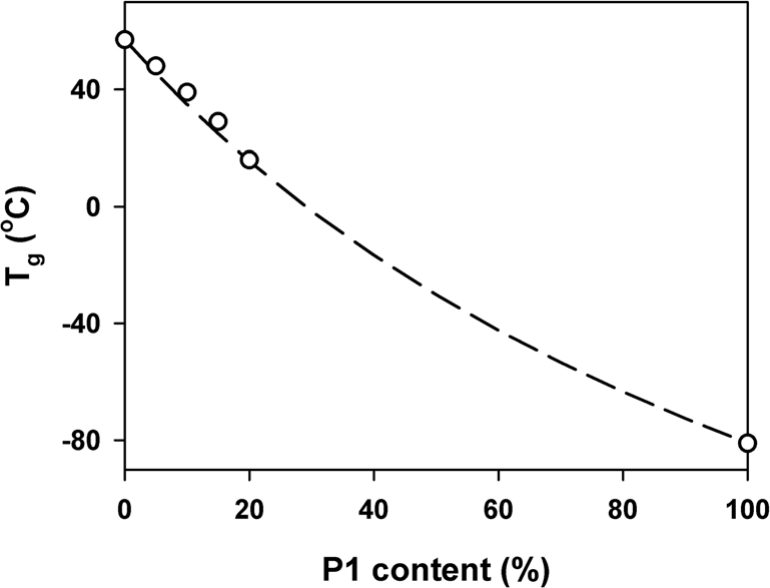
Dependence of the glass transition temperature of PLA/P1 blends determined from the DSC thermograms on blend composition. *Dashed line*: prediction based on the Fox equation. *Symbols*: experimental data

*T*_g_ of binary miscible blends is often expressed by the empirical Fox equation [42]:

1 $$ {T_{\mathrm{g}}}^{-1}\kern0.5em =\kern0.75em {w}_1{T_{\mathrm{g}1}}^{-1}\kern0.5em +\kern0.75em {w}_2{T_{\mathrm{g}2}}^{-1} $$

where *w*_1_ and *w*_2_, *T*_g1_ and *T*_g2_ are the weight fractions and *T*_g_s of the blend components. Figure 5 shows that the *T*_g_ of homogeneous PLA/P1 blends followed the Fox equation. Assuming the same for the continuous phase of PLA/P2 blends and taking into account that *T*_g_ of pure P2 was at −84 °C, it can be calculated based on Eq. 1 that the continuous phase of PLA/P2-15 and PLA/P2-20 contained about 3 wt% of P2 whereas the rest, that is, 12 wt% and 17 wt%, respectively, formed the droplets.

**Fig. 6:**
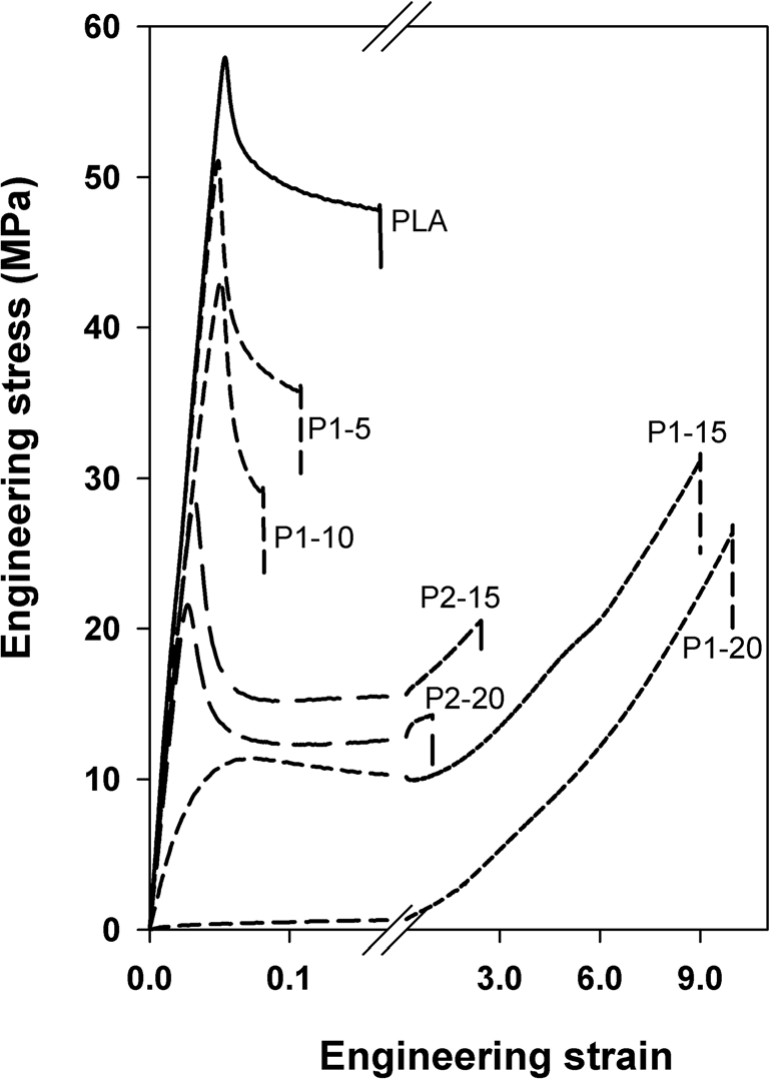
Tensile stress–strain behavior of PLA, PLA/P1, and PLA/P2 blends

The stress–strain dependencies of the blends are plotted in Fig. 6, whereas the average values of yield stress *σ*_y_, stress at break *σ*_b_, elongation at break *ε*_b_, and 2 % secant modulus of elasticity are collected in Table 2. Neat PLA yielded at *σ*_y_ of 58 MPa and fractured early at *ε*_b_ of 0.15 and *σ*_b_ of 48 MPa. *σ*_y_ and *σ*_b_ of PLA/P1 blends, ranging from 0.4 to 50 MPa, and from 25 to 36 MPa, respectively, were below those of neat PLA and decreased with increasing P1 content. The stress–strain dependencies of PLA/P1-5 and PLA/P1-10 were featured by distinct yield points at relatively high *σ*_y_ above 40 MPa. These blends fractured at small *ε*_b_ below that of neat PLA. Crazing in the gauge regions of deformed specimens was visible even to the naked eye. Increase of P1 content in the blends to 15–20 wt% caused a dramatic improvement of ductility reflected in a drop of *σ*_y_ and improvement *ε*_b_ to 9–10. PLA/P1-20 specimens deformed uniformly without necking and any signs of localized plastic deformation or cavitation related phenomena, in an elastomer-like manner. Owing to the lack of a distinct yield point, *σ*_y_ of PLA/P1-20 had to be determined with 2 % offset and was equal to 0.4 MPa. The intense strain hardening in PLA/P1-15 and PLA/P1-20 resulted in *σ*_b_ of about 29 and 25 MPa, respectively. Appearance of strain hardening is a sign of very significant plastic deformation of the amorphous phase and straining of the chain entanglement network.

**Table 2:** Tensile properties of PLA, PLA/P1, and PLA/P2 blends: average and standard deviation (in brackets) values of yield stress, *σ*_y_, stress, *σ*_b_, and elongation, *ε*_b_, at break and 2 % secant modulus of elasticity *E*_s_

Sample code	*σ*_y_ (MPa)	*σ*_b_ (MPa)	*ε*_b_	*E*_s_ (MPa)
PLA	57.8 (0.8)	48.2 (0.8)	0.15 (0.05)	1,200 (30)
PLA/P1-5	50.4 (0.5)	36.1 (0.7)	0.11 (0.02)	1,130 (20)
PLA/P1-10	42.7 (0.4)	32.4 (5.7)	0.08 (0.04)	1,010 (30)
PLA/P1-15	11.6 (0.3)	28.8 (2.7)	8.8 (0.6)	330 (18)
PLA/P1-20	0.4 ^a^ (0.1)	25.1 (1.1)	10.0 (0.4)	30 (5.5)
PLA/P2-15	28.8 (0.2	20.1 (0.9)	2.3 (0.3)	730 (30)
PLA/P2-20	22.5 (0.8)	14.5 (0.5)	1.0 (0.4)	490 (22)

^a^Yield stress determined with 2 % offset

*E*_s_ of PLA/P1 blends was lower than that of neat PLA, 1,200 MPa, and diminished with increasing plasticizer content to 30 MPa for PLA/P1-20. Such low value of *E*_s_ is consistent with the elastomeric-like behavior of this blend.

Also the addition of P2 improved markedly the ductility of PLA. PLA/P2 blends exhibited a pronounced yield at *σ*_y_ being approximately half of that of neat PLA. *ε*_b_ was for 2.3 PLA/P2-15 but diminished to 1 for PLA/P2-20. *E*_s_ values of the blends were lower than that of neat PLA but exceeded *E*_s_ of PLA/P1 blends with the same plasticizer contents.

### Mechanism of plastic deformation of PLA/P2 blends

**Fig. 7:**
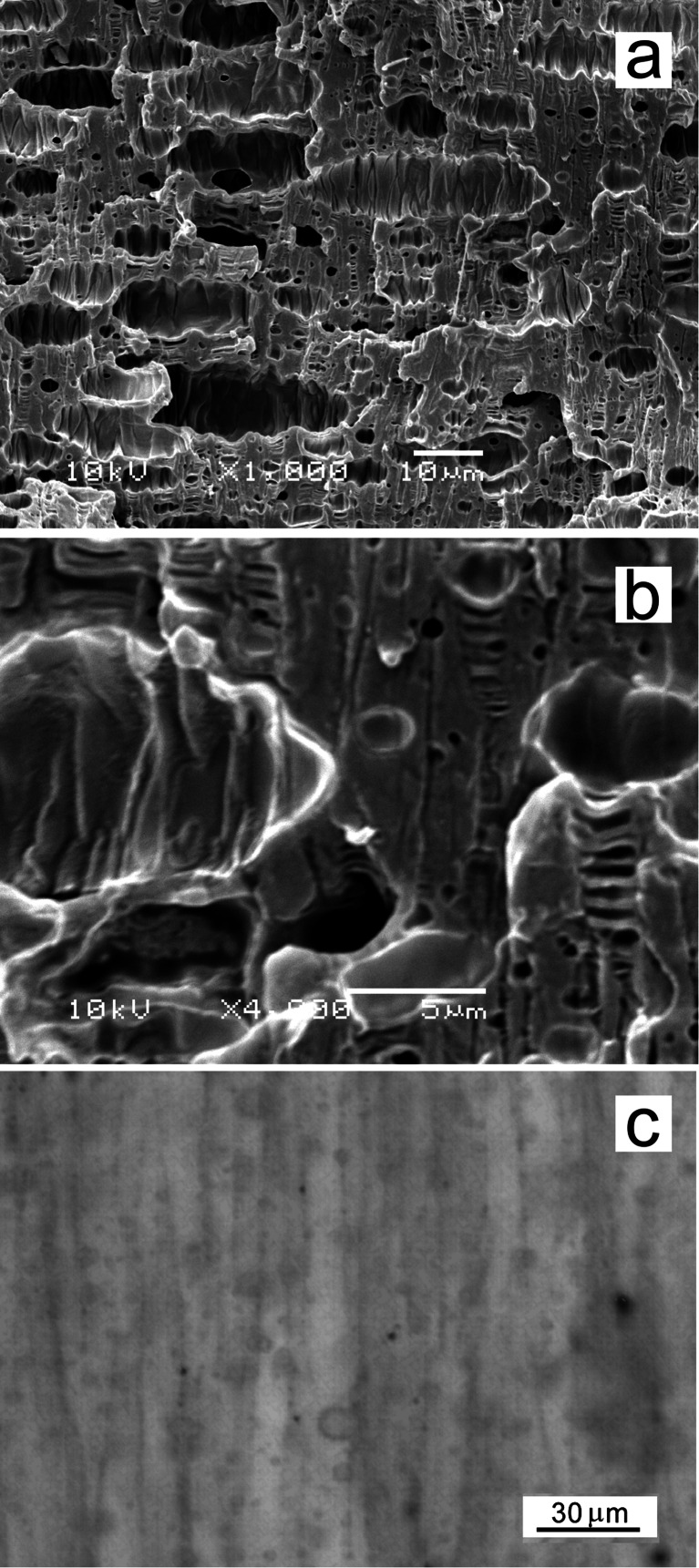
SEM (**a**, **b**) and PLM (**c**) micrographs of the gauge zone of PLA/P2-15 tensile specimen, which fractured at a strain of 2.6 and for SEM was cryo-fractured parallel to the drawing direction. The drawing direction, horizontal

Although the decrease of *T*_g_ of PLA/P2 blends to 48–50 °C undoubtedly increased segmental mobility of PLA chains, it cannot be the only reason of the improved ductility of these materials because plasticized PLA becomes ductile when its *T*_g_ decreases to at least 35 °C [8]. PLA/P1-5 blend with *T*_g_ at 48 °C exhibited relatively high *σ*_y_, and *ε*_b_ even smaller than that of neat PLA. During drawing, an intense stress whitening in both PLA/P2 blends was observed from the very beginning of plastic deformation. SEM and PLM micrographs of PLA/P2-15 specimen, which fractured at elongation of 2.6 and for SEM was cryo-fractured parallel to the drawing direction, in Fig. 7, demonstrate a heavily crazed matter with a network of crazes. The crazes were obviously originated and terminated by inclusions, although empty holes where P2 was accumulated are seen rather than the inclusions. The holes are elongated in the drawing direction and have undulant surface, resulting most probably from the localization of plastic deformation in crazes and also from the strong post-break shrinkage of the tensile specimen, which reduced the strain to about half of that at break. As reported so far, toughening of PLA by blending with immiscible polymers was associated mainly with cavitation either in the dispersed particles [22] or at the particle–matrix interface [21], which facilitated shear yielding of PLA matrix. In Kowalczyk and Piorkowska [22], poly(1,4-*cis*-isoprene) particles initiated crazes in PLA matrix, but cavitation inside the particles promoted shear yielding. In the present study, neither SEM nor PLM provided evidence of shear bands in the deformed PLA/P2-15 blend; all crazes were well aligned perpendicular to the drawing direction. Moreover, the plastic deformation, although concentrated in the gauge region, proceeded without necking. It was also noticed that crazing occurred at the very beginning of the deformation process. According to refs. [43, 44], pools of plasticizing diluents dispersed in glassy polymer can promote crazing because the diluent from pools that are tapped by an advancing craze spreads along the craze border between the craze fibrils, driven by capillary forces, and sorption of the diluent into plastically deforming base region of the craze fibrils occurs, enhanced by the plastic flow of polymer [45]. The resulting local plasticization decreases the craze flow stress and therefore facilitates propagation of crazes. In PLA/P2 blends, the liquid inclusions of P2 not only initiated crazes but also promoted their propagation in matrix via such local plasticizing effect. This mechanism could act at the very beginning of craze formation because, unlike in Argon [43] and Piorkowska et al. [44], the inclusions initiating crazes were liquid pools themselves. As a result, the crazing prevailed and shear yielding did not occur. The drainage of liquid modifier into crazes left emptied holes in matrix which could easily elongate in the drawing direction. Gebizlioglu et al. [46] observed that to toughen polystyrene, liquid pools of low-molar-mass polybutadiene had to be of submicron size. The ductile PLA/P2-15 blend contained much larger liquid inclusions. However, an increase of P2 content from 15 to 20 wt% had a detrimental effect on *ε*_b_, most possibly because of an excessive increase in the inclusion size, similarly as reported for other PLA-based blends, e.g. [22], as too-large inclusions can promote early fracture. Nevertheless, PLA/P2-15 blend exhibited good drawability while maintaining the relatively high *T*_g_, yield strength and stiffness, typical of a rubber toughened PLA rather than of a plasticized PLA. In contrast to other phase-separated PLA-based blends, both PLA/P2 blends were transparent.

### Aging of PLA/P1-20 blend

**Fig. 8:**
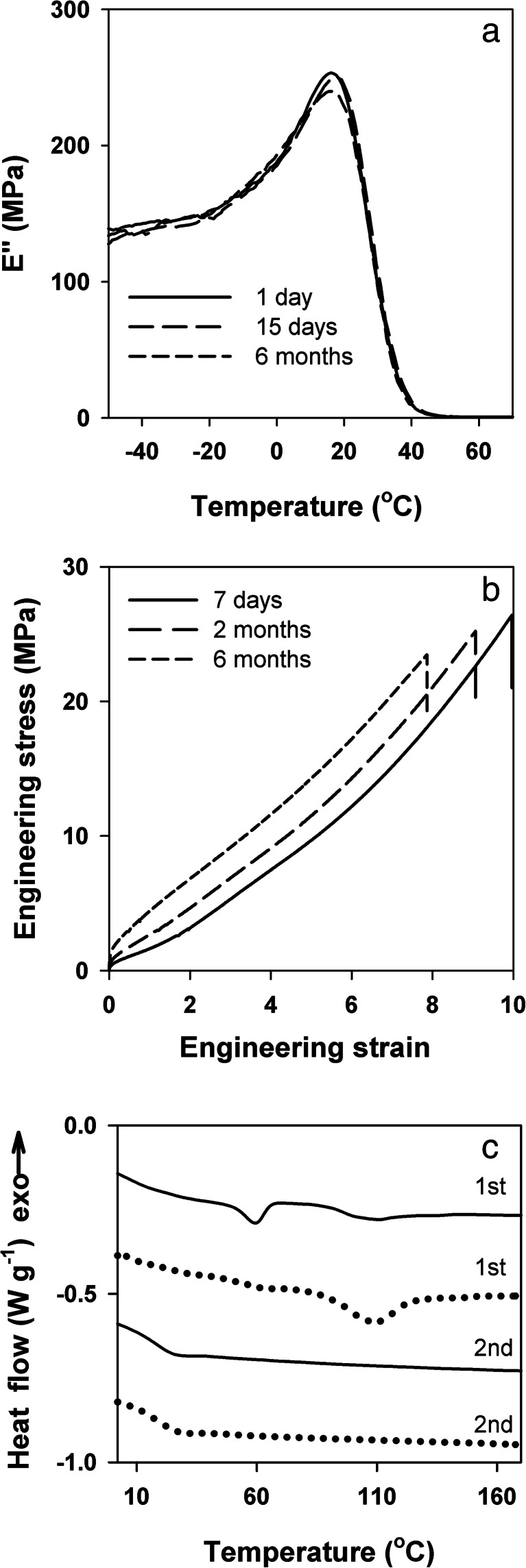
Effect of aging at room temperature on loss modulus *E*'' temperature dependence (**a**), tensile properties (**b**), and thermal properties (**c**) of PLA/P1-20 blend. **c** Shows the first and the second DSC heating thermograms of the blend after aging for 6 months at room conditions (*solid line*) and after aging for 28 days at 35 °C (*dotted line*). Thermograms shifted vertically for clarity

According to Hu et al. [9, 10], the most intense aging phenomena occur in PLA/PEG blends with *T*_g_ below ambient temperature and slow down when their *T*_g_ increases. Only the homogeneous PLA/P1-20 blend had *T*_g_ below room temperature, at 16 °C. The temperature dependencies of *E*'' measured after 1 day, 15 days, and 6 months of aging at ambient temperature are plotted on a linear scale in Fig. 8a for temperatures ranging from −50 to 70 °C. The curves did not exhibit any marked differences; after 6 months of aging, the *E*'' peak temperature was 16 °C, the same as for unaged blend. Nevertheless, as illustrated in Fig. 8b, aging caused gradual changes in the tensile behavior. After 6 months, *σ*_y_ of PLA/P1-20 increased to 1.4 MPa, whereas *σ*_b_ and *ε*_b_ decreased to approximately 23.5 MPa and 8, respectively. *E*_s_ also increased, to 60 MPa. The DSC heating thermogram recorded after 6 months of aging presented in Fig. 8c exhibited a small melting peak at 60 °C and a broad endotherm ending near 140 °C. A complex melting behavior of low optical purity PLAs, with multiple melting peaks, was reported previously by others [47]. The melting enthalpy of the aged PLA/P1-20 was about 7 Jg^-1^, which corresponds to the crystallinity level of 7 wt% if the enthalpy of fusion for the alpha orthorhombic form of PLA, 106 J g^−1^, is assumed [47]. The presence of that amount of stiff crystalline phase of PLA was obviously responsible for the change in mechanical properties of PLA/P1-20. In general, crystallization increases plasticizer content in the amorphous phase and can therefore affect *T*_g_, but the crystallinity level in aged PLA/P1-20 was too small to cause a noticeable change in *T*_g_ of the blend. Moreover, the blend remained transparent. It can be also seen that the second heating thermogram of the aged blend does not differ from the first heating thermogram of the unaged blend, as shown in Fig. 1, indicating that no irreversible changes in the thermal behavior of the material occurred upon aging.

Despite the small morphology changes upon aging, the tensile behavior of PLA/P1-20 remained elastomer-like with the low *T*_g_, small *σ*_y_, large *ε*_b_, and strong strain-hardening leading to high value of *σ*_b_. It can be concluded that, after 6 months of aging, the blend retained very good ductility, flexibility, and also transparency.

During aging at 35 °C PLA in PLA/P1-20 crystallized faster than at room temperature. The first heating thermogram recorded after 7 days (not shown) and 4 weeks (Fig. 8c) exhibited broad melting endotherms. The melting enthalpy after 7 days of aging was about 13 J g^−1^ which corresponds to the crystallinity of 12 wt%. Longer aging did not increase the crystallinity of the blend any further; after the next 21 days, it was at the same level. It can be seen that the first heating thermogram of the blend aged at 35 °C differs from that of the blend aged at room temperature only above 50 °C, where the melting of the crystalline phase occurs. The second heating thermogram of PLA/P1-20 aged at 35 °C differs neither from that of the blend aged at room temperature nor from that of the unaged blend. It appears that, although aging at 35 °C accelerated crystallization of PLA, it did not result in any irreversible changes in the thermal behavior of the blend, similar to aging at room conditions.

## Conclusions

Novel blends of polylactide with ethylene glycol derivatives engrafted POSS were prepared by simple blending and studied. The first of them, P1, was a commercial POSS with PEG methyl ether arms, whereas the second, P2, was synthesized by hydrosilylation reaction of 3-[2-(2-ethoxyethoxy)ethoxy]-propene-1 with octakis(dimethylsiloxy) octasilsesquioxane [HSi(CH_3_)_2_O]_8_(SiO_1.5_)_8_.

Both types of hybrids efficiently modified the mechanical properties of PLA. POSS substituted with PEG methyl ether arms, −CH_2_CH_2_(OCH_2_CH_2_)_m_OCH_3_ (average *m* of 13.3), was well miscible with PLA, despite its relatively large molar mass (*M*_w_ of 9,500 g mol^−1^), and acted as a plasticizer, efficiently decreasing *T*_g_; the blends with up to 20 wt% of the plasticizer were transparent and exhibited single glass transitions. The blends with 15 and 20 wt% of the plasticizer having *T*_g_ of 29 and 16 °C, respectively, were ductile. PLA/P1-20 was transparent and elastomeric-like material with low *σ*_y_ of 0.4 MPa and *ε*_b_ of 10 (1,000 %), and *E*_s_ of only 30 MPa, two orders magnitude lower that that of neat PLA. Although during 6 months aging at room temperature a small crystallinity level of 7 wt% developed in this blend, resulting in an increase in *σ*_y_ and *E*_s_, the elastomeric-like behavior was retained. The blend remained homogeneous and retained its good drawability as well as flexibility after 6 months of aging at room temperature: *E*_s_ well below 100 MPa, *σ*_y_ below 2 MPa, and a large *ε*_b_ of 8 (800 %). Increase of aging temperature to 35 °C accelerated crystallization of PLA in the blend, but the crystallinity level reached was small, about 12 wt%. We note that using PLA with a higher d-lactide content, unable to crystallize, will allow achieving even better stability.

Miscibility of POSS with relatively short arms of –OSi(CH_3_)_2_CH_2_CH_2_CH_2_(OCH_2_CH_2_)_2_OCH_2_CH_3_ comprising –OSi– with PLA was worse. PLA/P2 blends contained distinct inclusions of P2-rich phase, exhibiting a separate glass transition below −70 °C. T_g_ of the continuous phase in PLA/P2-15 and PLA/P2-20 was only several degrees below that of neat PLA. PLA/P2-15 exhibited better drawability than PLA/P2-20. It fractured at *ε*_b_ of about 2.3 (230 %) and exhibited *σ*_y_ and *E*_s_ approximately two times smaller than those of neat PLA. Such mechanical behavior is typical of rubber-toughened PLA rather than of plasticized PLA. Owing to the inability of P2 to crystallize, the inclusions were liquid. The liquid inclusions initiated crazes and also promoted their growth, thus toughening the material. Unlike majority of PLA-based phase-separated blends, PLA/P2 blends were transparent.

## Appendix

Synthesis of octakis{3-[2-(2-ethoxyethoxy)ethoxy]-propyldimethylsiloxy}-octasilsesquioxane (P2)

### Reagents

Commercially available octakis (dimethylsiloxy)octasilsesquioxane [HSi(CH_3_)_2_O]_8_(SiO_1.5_)_8_ was obtained from Hybrid Plastics Inc. and used without further purification. Allyl bromide (99 % Reagent Plus, Aldrich), sodium hydroxide (micropills, pure p.a. POCh S.A.), and di(ethylene glycol) ethyl ether (>99 %, SAFC) were used as received.

The hydrosilylation catalyst, a platinum tetramethyldivinyldisiloxane complex (Karstedt’s catalyst) (2 % solution in xylene, low color), was purchased from ABCR GmbH. Toluene, which was used as a solvent in the hydrosilylation reaction, was carefully dried according to the literature procedures [48] and distilled prior to use. Dichloromethane and methanol (both of analytic grade) were used as received from POCh S.A.

### Analysis and general methodology

Liquid-state ^1^H NMR spectra of precursors and condensed soluble materials were recorded in CDCl_3_ as a solvent on DRX-500 MHz spectrometer. Gas chromatography (GC) analysis was performed on Hewlett-Packard 6890 chromatograph fitted with a 30-m capillary column HP-1 HP 190592–023 and equipped with a thermal conductivity TDC detector. The carrier gas was helium, and the flow rate was 5 mL min^−1^. The detector and injector temperatures were 250 °C. The temperature was changed from 60 to 240 °C at the rate of 10 °C min^−1^.

### Preparation of 3-[2-(2-ethoxyethoxy) ethoxy-propene-1

The 40.0 g (1.0 mol) of sodium hydroxide and 90 mL (0.67 mol) of 2-(2-ethoxyethoxy) ethanol (EtOCH_2_CH_2_OCH_2_CH_2_OH) were placed in a three-necked flask equipped with a dropping funnel, a thermometer, a condenser, and a tube with CaCl_2_. The mixture was stirred vigorously for 15 min at room temperature with a magnetic stirrer, and it turned yellow after that time. Allyl bromide (102 mL, 1.17 mol) was placed in the dropping funnel and added slowly to the mixture. The temperature in the flask did not exceed 50 °C. Once the addition of allyl bromide was finished, the reaction mixture was stirred at room temperature for another 16 h. The mixture was filtered to remove solids (NaBr and unreacted NaOH). The filtrate was collected and diluted with diethyl ether (200 mL). The solution was washed with distilled water until neutral pH was obtained. The aqueous fractions were collected and washed with dichloromethane (4 × 100 mL). Both organic fractions were dried over MgSO_4_ overnight. After filtration, the fractions were combined, and the volatiles were removed under reduced pressure. The residue was dried at room temperature under vacuum (1 × 10^−1^ mm Hg), to give 98.6 g of pure 3-[2-(2-ethoxyethoxy) ethoxy]-propene-1 as indicated by GC analysis (the reaction yield 84.5 %).

### Preparation of octakis {3-[2-(2-ethoxyethoxy)ethoxy]-propyldimethylsiloxy}-octasilsesquioxane

[HSi(CH_3_)_2_O]_8_(SiO_1.5_)_8_ (20.05 g, 0.0179 mol) and 3-[2-(2-ethoxyethoxy)ethoxy]-propene-1 (25.0 g, 0.1435 mol) were placed in a flask equipped with a condenser, an inlet of argon, and a magnetic stirrer and dissolved in dry toluene (170 mL). The solution of Pt(0)-divinyltetramethyldisiloxane (0.5 mL, 2 % Pt) was added to the stirred mixture at room temperature. After 1 h, the temperature was increased to 80 °C, and the mixture was kept at this temperature for 48 h. The progress of addition of SiH to alkene bonds was followed with ^1^H NMR spectroscopy. Once it was complete, the volatiles were removed under reduced pressure. The polymeric product was collected and dried to constant weight at room temperature under vacuum (0.1 mm Hg), to give 42.83 g of pure octakis{3-[2-(2-ethoxyethoxy)ethoxy]-propyldimethylsiloxyl}-octasilsesquioxane (the reaction yield 95.1 %).

^1^H NMR (CDCl_3_) δ (ppm)—0.15 s SiMe_2_ (48H), 0.5 m OCH_2_CH_2_CH_2_ (16H), 1.2 t CH_3_CH_2_O (24H), 1.6 m OCH_2_CH_2_CH_2_ (16H), 3.4 m OCH_2_CH_2_CH_2_ (16H), 3.5 m CH_3_CH_2_O (16H), 3.6 m OCH_2_CH_2_O (64H).

## References

[1] Pluta M, Galeski A (2002). J Appl Polym Sci.

[2] Perego G, Cella GD, Bastioli C (1996). J Appl Polym Sci.

[3] Labrecque LV, Kumar RA, Dave V, Gross RA, McCarthy SP (1997). J Appl Polym Sci.

[4] Ljungberg N, Wesslen B (2002). J Appl Polym Sci.

[5] Yang JH, Shen Y, He WD, Zhang N, Huang T, Zhang JH, Wang Y (2013). J Appl Polym Sci.

[6] Sheth M, Kumar RA, Dave V, Gross RA, McCarthy SP (1997). J Appl Polym Sci.

[7] Jacobsen S, Fritz HG (1999). Polym Eng Sci.

[8] Baiardo M, Frisoni G, Scandola M, Rimelen M, Lips D, Ruffieux K, Wintermantel E (2003). J Appl Polym Sci.

[9] Hu Y, Rogunova M, Topolkaraev V, Hiltner A, Baer E (2003). Polymer.

[10] Hu Y, Hu YS, Topolkaraev V, Hiltner A, Baer E (2003). Polymer.

[11] Kulinski Z, Piorkowska E (2005). Polymer.

[12] Kulinski Z, Piorkowska E, Gadzinowska K, Stasiak M (2006). Biomacromolecules.

[13] Piorkowska E, Kulinski Z, Galeski A, Masirek R (2006). Polymer.

[14] Kowalczyk M, Pluta M, Piorkowska E, Krasnikova N (2012). J Appl Polym Sci.

[15] Okamoto K, Ichikawa T, Yokohara T, Yamaguchi M (2009). Eur Polym J.

[16] Ljungberg N, Wesslen B (2004). J Appl Polym Sci.

[17] Ljungberg N, Wesslen B (2005). Biomacromolecules.

[18] Pluta M, Paul MA, Alexandre M, Dubois P (2006). J Polym Sci Part B-Polym Phys.

[19] Hassouna F, Raquez JM, Addiego F, Dubois P, Toniazzo V, Ruch D (2011). Eur Polym J.

[20] Hassouna F, Raquez JM, Addiego F, Toniazzo V, Dubois PH, Ruch D (2012). Eur Polym J.

[21] Jiang L, Wolcott MP, Zhang J (2006). Biomacromolecules.

[22] Kowalczyk M, Piorkowska E (2012). J Appl Polym Sci.

[23] Dong W, Cao X, Li Y (2014) Polym Intern 63:1094–1100

[24] Argon AS, Cohen RE, Gebizlioglu OS, Schwier CE (1983) Adv Polym Sci 52/53:275–334

[25] Lazzeri A, Bucknall CB (1993). J Mater Sci.

[26] Shen J, Zheng S (2006). J Polym Sci Part B-Polym Phys.

[27] Maitra P, Wunder SL (2002). Chem Mater.

[28] Kim BS, Mather PT (2002). Macromolecules.

[29] Lee W, Ni S, Deng J, Kim BS, Satija SK, Mather PT, Esker AR (2007). Macromolecules.

[30] Mya KY, Pramoda KP, He CB (2006). Polymer.

[31] Zhang H, Kulkarni S, Wunder SL (2007). J Phys Chem B.

[32] Mya KY, Li X, Chen L, Ni XP, Li J, He CB (2005). J Phys Chem B.

[33] Lestel L, Cheradame H, Boileau S (1990). Polymer.

[34] Ryu HS, Kim DG, Lee JC (2010). Macrom Res.

[35] Knischka R, Dietsche F, Hanselmann R, Frey H, Mülhaupt R, Lutz PJ (1999). Langmuir.

[36] Wang D, Fredericks PM, Haddard A, Hill DJT, Rasoul F, Whittaker AK (2011). Polym Degrad Stab.

[37] Kim SK, Heo SJ, Koak JY, Lee JH, Lee YM, Chung DJ, Lee JI, Hong SD (2007). J Oral Rehabil.

[38] Punshon G, Vara DS, Sales KM, Kidane AG, Salacinski HJ, Seifalian AM (2005). Biomaterials.

[39] Kim KO, Kim BS, Kim IS (2011). J Biomater Nanobiotech.

[40] Wang DK, Varanasi S, Strounina E, Hill DJT, Symons AL, Whittaker AK, Rasoul F (2014). Biomacromolecules.

[41] Bailey FE, Koleske JV (1976). Poly(ethylene oxide).

[42] Olabisi O, Mobeson LM, Shaw MT (1979). Polymer–polymer miscibility.

[43] Argon AS (1999). J Appl Polym Sci.

[44] Piorkowska E, Argon AS, Cohen RE (1993). Polymer.

[45] Zhou QY, Argon AS, Cohen RE (2001). Polymer.

[46] Gebizlioglu OS, Beckham HW, Argon AS, Cohen RE, Brown HR (1990). Macromolecules.

[47] Sarasua JR, Prud’homme RE, Wisniewski M, Le Borgne A, Spassky N (1998). Macromolecules.

[48] Perrin DD, Armarego WLF (1980). Purification of laboratory chemicals.

